# Supplementary material for: Online search interest in long-term symptoms of coronavirus disease 2019 during the COVID-19 pandemic in Japan: Infodemiology study using the most visited search engine in Japan
Source: PLoS One. 2023 Nov 15;18(11):e0294261. doi: 10.1371/journal.pone.0294261 (PMC10650984; doi:10.1371/journal.pone.0294261)
Supplement: S1 Table — (PDF) [file pone.0294261.s001.pdf]

S1. Table. The explanation of Yahoo! Japan DS. INSIGHT functions

**1. How to use the basic screen**
(1) Period<br>-You can select any of the following: 1 day, 1 week, 1 month, 1 year, or the most recent 1-year period.
(2) Attributes<br>-You can set up search conditions for keywords by gender, age, and region.
(3) Narrow down the search by recently searched conditions<br>-Up to 100 keywords, time periods, and search criteria entered will be displayed as history. You can use this function when you want to perform the same search again.
(4) Co-occurrence keyword map/ranking<br>-You can switch between a map of keywords searched together with the search keyword and a ranking of keywords that include the search keyword.
**2. Check the information of the selected keyword**
(1) Keyword comparison<br>-You can compare information on multiple keywords.
(2) Attribute Graph<br>-Displays search volume, gender ratio, and age ratio of the keywords searched.
(3) Percentage by region<br>-You can switch and display the search volume and characteristic level of the selected keyword by prefecture.
(4) Search Trends<br>-Two types of graphs are displayed: one for the trends in search volume over the past 4 years, and the other for a comparison of search volume trends over a 4-year period.
(5) Time-series keywords<br>-The keywords that users searched for before and after the target keyword are displayed.

Source: Yahoo Japan Data Solution DS. INSIGHT
