# Supplementary material for: Online search interest in long-term symptoms of coronavirus disease 2019 during the COVID-19 pandemic in Japan: Infodemiology study using the most visited search engine in Japan
Source: PLoS One. 2023 Nov 15;18(11):e0294261. doi: 10.1371/journal.pone.0294261 (PMC10650984; doi:10.1371/journal.pone.0294261)
Supplement: S2 Table — (PDF) [file pone.0294261.s002.pdf]

S2 Table. Annual number of searches for long-COVID by prefecture

Prefecture	2020	2021	2022
	Number of searches for long-COVID (75,900)	Number of searches for long-COVID (164,800)	Number of searches for long-COVID (594,000)
Hokkaido	2,500	5,400	21,100
Aomori	260	520	2,600
Iwate	260	450	1,900
Miyagi	870	1,700	6,800
Akita	220	280	1,900
Yamagata	250	440	2,200
Fukushima	440	770	3,700
Tokyo	11,200	26,700	86,200
Kanagawa	4,500	11,100	36,800
Saitama	3,700	9,300	33,000
Chiba	2,800	6,800	22,400
Ibaraki	990	2,000	7,700
Tochigi	530	1,200	4,800
Gunma	540	1,000	4,400
Yamanashi	210	420	1,900
Niigata	470	820	4,000
Nagano	460	1,500	9,000
Toyama	470	890	4,200
Ishikawa	470	1,000	4,500
Fukui	270	550	2,800
Aichi	5,000	11,100	45,700
Gifu	910	1,900	8,500
Shizuoka	1,700	3,700	15,200
Mie	900	1,600	7,100
Osaka	6,500	16,700	61,000
Hyogo	2,400	6,000	20,500
Kyoto	1,200	2,900	10,400
Shiga	610	1,200	5,400
Nara	560	1,200	4,700
Wakayama	330	520	2,400
Tottori	120	200	980
Shimane	160	180	1,100
Okayama	610	1,200	5,000
Hiroshima	880	1,900	7,800
Yamaguchi	350	600	2,400
Tokushima	220	450	1,800
Kagawa	270	490	2,400
Ehime	340	610	2,900
Kochi	150	290	1,300
Fukuoka	1,500	4,000	15,000
Saga	140	240	1,300
Nagasaki	220	420	2,000
Kumamoto	320	580	2,600
Oita	270	500	1,900
Miyazaki	230	380	2,300
Kagoshima	170	370	1,900
Okinawa	290	870	2,900

Source: Yahoo Japan Data Solution DS. INSIGHT
