# Supplementary material for: Online search interest in long-term symptoms of coronavirus disease 2019 during the COVID-19 pandemic in Japan: Infodemiology study using the most visited search engine in Japan
Source: PLoS One. 2023 Nov 15;18(11):e0294261. doi: 10.1371/journal.pone.0294261 (PMC10650984; doi:10.1371/journal.pone.0294261)
Supplement: Attachment — Submitted filename: Response-to-Reviewer_Ishizuka.docx [file pone.0294261.s004.docx]

October 27, 2023

Dr. Omar Enzo Santangelo

**Title:** Online Search Interest in long-term symptoms of coronavirus disease 2019 During the COVID-19 Pandemic in Japan: Infodemiology Study Using the Most Visited Search Engine in Japan

**Reference number:** PONE-D-23-28136.R1

Dear Dr. Omar Enzo Santangelo,

Thank you for your e-mail of October 13, 2023, regarding our manuscript, “**Online Search Interest in long-term symptoms of coronavirus disease 2019 During the COVID-19 Pandemic in Japan: Infodemiology Study Using the Most Visited Search Engine in Japan**”, and for the valuable comments of the reviewers. I have attached our revised manuscript, as well as a point-by-point response to the reviewers’ comments.

We hope that the revised manuscript contains suitable responses to the comments, and we think that it has been significantly improved over the previous submission. We trust that our manuscript is now suitable for publication in PLOS ONE.

Thank you in advance for your kind consideration of our work.

Sincerely yours,

Kosuke Ishizuka, MD, PhD

Department of General Medicine, Yokohama City University School of Medicine

3-9 Fukuura, Kanazawa-ku, Yokohama-city, Kanagawa pref. Japan

Tel. +81-45-787-2800

Fax. +81-45-350-2728

E-Mail: e103007c@yokohama-cu.ac.jp

**RESPONSES TO REVIEWER 1:**

We wish to express our appreciation to the reviewer for insightful comments that have helped us to improve our paper. We hope that the revised manuscript contains suitable responses to the comments, and we think that it has been significantly improved over the previous submission. We trust that our manuscript is now suitable for publication in PLOS ONE.

## **NOTE**

We highlighted changes of significant issues in yellow (please see “Revised Manuscript with Track Changes”).

**Comments:**

I read your revised paper throughout, and it has definitely improved. I still have one minor comment:

**Response:**

Thank you for your general comments.

Per your comments and amendments, we have revised our manuscript.

1) Reference numbers 3 and 14: Are these books or what kind of references? Please add online links if available.

**Response:**

Thank you for pointing it out.

We added the References number 3-6, so Reference number 3 in the previous manuscript corresponds to Reference number 7 in the revised manuscript, and Reference number 14 in the previous manuscript corresponds to Reference number 18 in the revised manuscript.

Reference number 7 in the revised manuscript is an online article. We added the online link.

In addition, Reference number 18 in the revised manuscript is a book as following link; https://books.google.co.jp/books/about/ICPC_2.html?id=Wr_3OqVxypMC&redir_esc=y. We reconfirmed that the reference is correctly described according to the PLOS ONE submission guidelines.

**Changes:**

- **References** (References; lines 340 to 454.)

## **Others**

We also indicated other minor corrections in yellow markers (please see “Revised Manuscript with Track Changes”).

We thank the reviewer for such pertinent comments. We hope that the revised manuscript contains suitable responses to the comments, and we think that it has been significantly improved compared to the previous submission. We trust that our manuscript is now suitable for publication in PLOS ONE.

**RESPONSES TO REVIEWER 2:**

We wish to express our appreciation to the reviewer for insightful comments that have helped us to improve our paper. We hope that the revised manuscript contains suitable responses to the comments, and we think that it has been significantly improved over the previous submission. We trust that our manuscript is now suitable for publication in PLOS ONE.

## **NOTE**

We highlighted changes of significant issues in yellow (please see “Revised Manuscript with Track Changes”).

**Comments:**

Nice job incorporating reviewers' comments. Two items:

**Response:**

Thank you for your general comments.

Per your comments and amendments, we have revised our manuscript.

In addition, we have obtained help from a professional editor to make it more understandable, and free from awkward expressions. Please find a certificate of proofreading in English attached.

We answered each of the questions you posed in the revised manuscript.

1) "nineth" is misspelled.

**Response:**

Thank you for pointing it out. Along with your comments, we revised the description.

**Changes:**

- Ninth, because search data was only available after 2020, when the pandemic began, we could not determine whether the number of searches represent an absolute increase in the number of searches for these symptoms or simply fluctuations in the number of searches over time during the period when COVID-19 was prevalent. (Limitations; lines 320 to 324.)

2) Consider including when Long-Covid was first reported. This is helpful to know for any temporal analysis of this term.

**Response:**

As you have indicated, we included when long-COVID was first reported as follows:

**Changes:**

- The term “long-COVID” first entered public discourse on May 20, 2020, when Dr. Elisa Perego shared #longcovid on Twitter, now known as X [3-5]. A hashtag (#) is a concept-labelling tool that encourages public sharing and worldwide dissemination of the discussed concept by grouping conversations around it. Initially, #longcovid referred to Perego's experience of prolonged “cyclical, multiphasic, and multisystem” symptoms of COVID-19 [5]. Twitter, with approximately 187 million users worldwide at the time, emerged as a platform for individuals with persistent COVID-19 symptoms to publicly share their experiences, symptoms, and concerns associated with living with the condition [5, 6]. Subsequently, on October 6, 2021, the World Health Organization (WHO) defined long-COVID as the onset of symptoms within 3 months after the onset of COVID-19 and persistence of symptoms for more than 2 months, excluding other diseases [7, 8]. (Introduction; lines 70 to 81.)

## **Others**

We also indicated other minor corrections in yellow markers (please see “Revised Manuscript with Track Changes”).

We thank the reviewer for such pertinent comments. We hope that the revised manuscript contains suitable responses to the comments and has been significantly improved compared to the previous submission. We trust that our manuscript is now suitable for publication in PLOS ONE.
